# Quantification of EGFR mutations in primary and metastatic tumors in non-small cell lung cancer

**DOI:** 10.1186/1756-9966-33-5

**Published:** 2014-01-08

**Authors:** Bing Wei, Ke Yang, Jiuzhou Zhao, Yuxi Chang, Zihui Ma, Bing Dong, Yongjun Guo, Jie Ma

**Affiliations:** 1 Department of Pathology, Henan Cancer Hospital, the Affiliated Cancer Hospital of Zhengzhou University, Zhengzhou 450008, Henan, China

**Keywords:** Non-small cell lung cancer, EGFR mutations, Primary tumors, Metastatic tumors, Quantification

## Abstract

**Background:**

EGFR mutation detection has been widely applied in the prediction of TKIs therapy in Non-Small Cell Lung Cancer (NSCLC). Metastatic tumors rather than primary tumors were usually assayed for those patients in advanced stages. Although the difference of EGFR mutation status in primary and metastatic tumors has been reported, the quantitative difference (ratio of mutated EGFR among total EGFR) in primary and metastatic tumors as well as in different sites of primary tumors was not clear.

**Methods:**

Genomic DNA in Formalin Fixed-Paraffin Embedded samples of primary and metastatic tumors of 50 NSCLC patients was extracted. Real-time fluorescent PCR was performed to quantify the EGFR mutation ratios.

**Results:**

The EGFR mutation ratios detected in different sites of primary tumors were highly concordant, whereas the EGFR mutation ratios in metastatic tumors were lower than those in primary tumors.

**Conclusions:**

Randomly chosen sample may reliably represent the type and ratio of mutations of EGFR in primary tumors. EGFR mutation ratios in primary tumors and metastatic tumors are different. If metastatic tumors are used for the detection of EGFR mutation, the sensitivity of the detection assay must be considered.

## Background

Epidermal growth factor receptor (EGFR) is a receptor tyrosine kinase encoded by the c-erb-B1 proto-oncogene. Multiple studies showed that the efficacy of tyrosine kinase inhibitors (TKIs) in the treatment of Non-Small Cell Lung Cancer (NSCLC) is highly correlated with EGFR mutation status in exon 18–21 [1-4].

EGFR mutations have been detected in 30-50% of NSCLC patients in China [5,6]. The detection methods include PCR-sequencing, Taqman real-time PCR, DHPLC, and SARMS [6-12]. For some of the NSCLC patients, especially those with metastatic cancer, the primary tumor specimen may not be available; therefore EGFR mutations in metastases are often analyzed. However, the molecular nature of the tumors may change during metastasis, and currently it is unclear whether the mutations detected in primary tumors correlate with those in metastases. It has been reported that EGFR mutations detected in metastases are 10-60% inconsistent with those in primary tumors [13,14]. It is worth noting that gefitinib has been reported to be beneficial for patients in which EGFR mutations were detected in metastases but not primary tumors [15]. However, since these studies used qualitative detection of EGFR mutations, it is impossible to quantitatively evaluate the abundance of EGFR mutations in the primary tumor and metastases.

Real-time fluorescent PCR detection of mutations is a straightforward method with high sensitivity and reliability. In this study, we used real-time PCR to quantitatively detect EGFR mutations in primary and metastatic tumors. Fifty Chinese NSCLC patients that harbor EGFR mutations in their primary tumors were identified. EGFR mutation status and abundance were compared among different areas of a primary tumor and its corresponding metastatic tumor of the same individual. Our study provides new insights on clinical interpretation of EGFR mutation status in different specimens.

## Methods

### Patients and Clinical Characteristics

From the patients who visited Henan Cancer Hospital between January 2010 and December 2012, those diagnosed with NSCLC by histological examination were tested for EGFR mutations, and 50 patients that were positive for EGFR mutations in the primary tumor samples were randomly selected for further evaluation. Their clinical and pathological characteristics are listed in Table 1. All study subjects never received TKI treatment before the study, and the formalin-fixed paraffin-embedded (FFPE) specimens were available for both the primary and metastatic tumors. Patients consented to tissue specimen collection prospectively, and the study was approved by the ethics committee of Henan Cancer Hospital, the Affiliated Cancer Hospital of Zhengzhou University.

**Table 1:** Clinical characteristics of 50 advanced NSCLC cases and the detection of EGFR mutations in primary tumors and metastases

	**No. cases**	**Mutation rates of primary tumor (%)**	**Mutation rates of metastases (%)**
Age			
>60	38	100	100
≤60	12	100	75
Gender			
Male	11	100	72.7
Female	39	100	100
Type			
Adenocarcinoma	49	100	95.9
Squamous cell carcinoma	1	100	0
Stage			
IIIB	28	100	89.3
IV	22	100	100
Smoking status			
Smoker	10	100	80
Non-smoker	40	100	97.5

### Clinical specimens

Pathological diagnosis was established as NSCLC by assessing the HE stained sections of formalin-fixed paraffin-embedded primary tumors. The tumor contents was >50% for slides prepared from primary tumors, and >20% for those from lymph node metastases. For each subject, four DNA samples corresponding to the two lateral regions and one center region of the primary tumor specimen, as well as one from lymph node metastases were prepared. For each sample, DNA was isolated from no less than 5 pieces of consecutive 5 μm slides of Formalin-fixed paraffin-embedded (FFPE) specimens that had been stored at room temperature for less than 5 years.

### Isolation of genomic DNA

Genomic DNA from the FFPE samples was isolated by using QIAamp DNA FFPE Tissue Kit (Qiagen) according to the manufacturer’s instructions. The DNA concentration was measured by UV spectrometer and adjusted to 20 ~ 50 ng/μl. DNA samples were stored at -20°C before use.

### Quantitative measurement of EGFR mutation ratio

45 types of EGFR mutations corresponding to hotspots in exons 18, 19, 20 and 21 were detected by using Human EGFR mutation quantitative PCR detection kit (ACCB, Beijing, China) according to the manufacturer’s instructions. Real-time PCR were performed on Stratagene Mx3000P PCR machine with the following settings: 95°C for 10 min, followed by 40 cycles of 95°C for 15 sec and 60°C for 1 min. The mutant and wild-type alleles were amplified separately, and the levels of each mutation in the sample were calculated by normalizing to standard curves. The mutation ratio was defined as [mutation ratio % = level of mutants/(level of mutants + level of wild type allele) × 100%].

### Statistical analysis

Statistical analysis was carried out using SPSS version 16.0 software (SPSS Inc., Chicago, IL, US). Fisher’s exact test was used to analyze whether the different categories had different positive rates. Kappa test was used to analyze whether the two sampling regions had consistent outcomes. Wilcoxon matched pairs test was used to compare the mutation ratios from the two regions. Two-sided p < 0.05 was considered statistically significant.

## Results

### EGFR mutations in primary tumors and metastases

Of the 50 cases of NSCLC that had EGFR mutations in primary tumors, exon 19 mutations (in-frame deletions only) were present in 28 cases (56%), and exon 21 (L858R point mutations only) mutations were detected in 22 cases (44%). Mutations in exon 19 and 21 were mutually exclusive and no multiple mutations were found. Of the metastases samples, 47 were positive for EGFR mutation (94% concordance with the detection in primary tumors), and exon 19 and exon 21 mutations were detected in 26 cases (55%, 93% concordance) and 21 cases (45%, 95% concordance), respectively. Notably, all cases presented the same mutation type in the matching primary and metastatic tumors. EGFR mutation detection and the clinical characteristics were listed in Table 1. Among the 50 subjects, only 3 (6%) had different test results for EGFR mutations in primary tumor and metastases, however, the difference was insignificant (P = 0.242) as analyzed by Fisher’s exact test.

### EGFR mutations at different sites of primary tumors of the same patient

We performed quantitative measurement of EGFR mutations at different sites of primary tumors (Table 2). The median mutation deviation for different primary sites (see footnote of Table 2 for the formula of calculation) was 18.3% (with a range of 0.0% ~ 54.3%), indicating that the results of the quantitative measurement of EGFR mutations in different sites of primary tumor in the same patient have a high level of concordance.

**Table 2:** Quantitative measurement of EGFR mutation ratios in 3 primary tumor sites and one metastases of the same patient

**ID**	**Mutation ratio (%) in different primary tumor sites**					**Mutation ratio (%) of metastases**
	**1**	**2**	**3**	**Median**	Deviation (%)*	
E001	85.9	91.1	80.1	85.9	12.8	<10
E002	39.1	25.9	44	39.1	49.8	41
E003	<10	<10	<10	<10	0.0	<10
E004	82.2	76.7	66.9	76.7	20.3	13.3
E005	43.9	40.5	45.4	43.9	11.3	41.2
E006	35.4	42.7	39.3	39.3	18.7	<10
E007	70.1	71.8	66.5	70.1	7.6	72.9
E008	79.8	85.1	88.9	85.1	10.8	28.1
E009	66.1	64.3	49.3	64.3	28.0	45.9
E010	54.2	83.6	77.6	77.6	40.9	15.9
E011	<10	<10	<10	<10	0.0	<10
E012	<10	<10	<10	<10	0.0	<10
E013	44.7	27.2	49.1	44.7	54.3	22.9
E014	55.5	64.3	66.6	64.3	17.9	31.2
E015	18.7	23.6	13.9	18.7	51.8	Negative
E016	37.6	45.2	38.2	38.2	18.8	<10
E017	23.8	28.9	23.9	23.9	20.0	30.4
E018	62.3	69.6	58.2	62.3	18.0	43.8
E019	<10	<10	<10	<10	0.0	<10
E020	<10	<10	<10	<10	0.0	<10
E021	48.6	47	40.2	47	18.6	25.3
E022	28.6	35.1	34.9	34.9	19.8	20.9
E023	38.9	31.7	30.9	31.7	23.6	35.9
E024	<10	<10	<10	<10	0.0	<10
E025	44.9	38.4	45.1	44.9	15.7	<10
E026	78.9	75.2	79.2	78.9	5.1	54.9
E027	<10	<10	<10	<10	0.0	Negative
E028	67.3	54.7	55.3	55.3	21.3	52.1
E029	56.3	45.4	47.5	47.5	21.9	<10
E030	24.8	29.1	32.7	29.1	27.4	15.9
E031	59.7	48.1	55.3	55.3	21.3	42.8
E032	31.8	34.9	41.1	34.9	25.9	25.8
E033	<10	<10	<10	<10	0.0	<10
E034	33.8	30.1	27.7	30.1	20.0	28.9
E035	42.2	38.1	45.1	42.2	16.7	40.2
E036	<10	<10	<10	<10	0.0	Negative
E037	54.7	48.4	47.1	48.4	15.2	<10
E038	18.3	28.7	22.2	22.2	45.1	14.9
E039	40.2	41.8	30.2	40.2	31.0	28.9
E040	38.4	45.2	43.2	43.2	16.1	<10
E041	58.3	51.9	48.3	51.9	18.9	45.5
E042	45.3	40.2	42.6	42.6	11.9	45.9
E043	<10	<10	<10	<10	0.0	<10
E044	51.1	55.3	44.8	51.1	20.8	32.7
E045	65.7	62.9	71.2	65.7	12.5	49.8
E046	28.9	29.8	33.1	29.8	13.7	19.6
E047	43.8	45.9	49.7	45.9	12.7	43.1
E048	67.3	63.2	52.2	63.2	24.8	33.8
E049	39.1	43.9	30.8	39.1	34.5	27.8
E050	28.9	21.8	21.6	21.8	30.3	22.5

*Mutation deviation (%) of primary tumors was defined as (Emax-Emin)/Emd × 100%, where Emax, Emin, and Emd are the maximum, minimum and median value of EGFR mutation ratios at different primary tumor sites. If all three mutation ratios in primary sites were below 10%, the deviation was calculated as 0%.

### Quantitative measurement of EGFR mutations in primary tumors and metastases of the same patient

Although the qualitative measurement of EGFR mutations in primary sites and metastases showed a high level of concordance (94%), the quantitative measurements had significant difference (Tables 2 and 3). The Kappa value of the two groups was 0.615 (P < 0.01), indicating that different sampling sites only had moderate concordance. Overall, the mutation ratios of metastases is significantly lower than those of primary tumors (P < 0.01) as analyzed by Wilcoxon matched pairs test.

**Table 3:** EGFR mutation ratios in primary tumor and metastases of the same patients

**EGFR mutation ratio**		**No. cases**	**%**
**Primary**	**Metastases**		
>10%	>10%	32	64%
<10%	<10% or negative	10	20%
>10%	<10% or negative	8	16%
<10%	>10%	0	0

## Discussion

NSCLC patients carrying EGFR mutations often benefit from TKI treatments with reduced sizes of primary tumors and metastases visualized by medical imaging. For a subset of patients, however, TKIs treatment only diminished the primary tumors but had no effect on metastases, and occasionally the metastases even became enlarged or more numerous over time. It raises the questions whether the abundance of EGFR mutations are different in different primary tumor sites, and whether the abundance and type of mutations are the same for primary tumors and metastases. Our study revealed the following characteristics of EGFR mutations.

First of all, although the mutation ratio in different primary tumor sites varied (the median value ranged from <10% to 85.9%) (Table 2), the deviation of the mutation ratio in different primary sites was limited (median was 18.3% with a range of 0.0% ~ 54.3%) (Table 2), indicating that different sites of primary tumor in the same patient have a high level of concordance. During the routine pathological evaluation of FFPE specimens of primary tumors, EGFR mutations were often tested only in one randomly chosen sample. Our study showed that when the area of cancerous cells were greater than 50%, a randomly chosen sample may reliably represent the type and ratio of mutations of EGFR in primary tumors.

Secondly, when the EGFR mutations were present in primary tumors, they could be detected in metastases with a high concordance regardless of the mutation ratios. The concordance of EGFR mutations in primary tumor and metastases is 94%, and that for mutation ratios is 84%. Moreover, different types of mutations, such as those in exon 19 and 21, were also identified with high concordances (93% and 95%, respectively), suggesting that the type of mutation did not affect the detection rates.

In addition, mutation detection is also affected by the proportion of cancerous cells in a sample. Therefore, for metastases with a lower number of cancerous cells, highly sensitive methods such as real-time PCR are highly recommended.

Moreover, in comparison to those in primary tumor sites, the mutation ratios in metastases were reduced and occasionally undetectable (16% samples had reduced or negative detection). These results suggest that the use of metastases specimens might generate false negative diagnosis for EGFR mutations that could have been present in primary tumors. The decreased EGFR mutation ratios in metastases suggest that EGFR mutations may not be essential for metastasis, which may underlie the lack of response to TKIs in metastases despite an positive outcome for the primary tumors. Notably, in this study we had one case of squamous cell carcinoma that harbors EGFR exon 19 mutation in the primary tumor, but the mutation was undetectable in metastases. It is unclear if it is due to the nature of squamous cell carcinoma.

In addition to the different pathological nature of primary tumor and metastases, the inconsistency in the identification of EGFR mutation may also be due to the sensitivity of the detection methods. For instance, Sanger sequencing may give a negative calling for samples with a mutation ratio of <10%, and therefore leads to low concordance for EGFR mutations in different samples of the same patients. Our study showed that by using quantitative real-time PCR, the positive identification of mutations in primary tumor and metastases had a 94% concordance, and for the quantitative measurement of mutation ratio the concordance was 84%. Hence, metastases specimens could be used for mutation assessment if the specimen for primary tumors is lacking, but the detection methods must be of high sensitivity. Recently, the abundance of mutations of predictive biomarkers, such as EGFR and KRAS, has drawn more attention [16-18]. It has been shown that the abundance of EGFR mutations predicts benefit from EGFR-TKI treatment for NSCLC [16]. Similarly, colorectal cancer patients with low abundance of KRAS mutation have been reported to benefit from EGFR antibody therapy [17]. Precise quantification of EGFR mutation abundance may become a trend in clinic to help with a better patient selection and better treatment strategies. To enable precise quantification of mutation ratio, real time PCR with a standard curve such as the method applied in this report serves as one of the optimal options.

In this study, only subjects with EGFR mutations in primary tumors were included, but it did not address the issues of positive mutation detection in metastases but negative in primary sites. Future studies should combine the prognosis data of the patients that received TKIs therapy and analyze the correlation between the quantitative measurement of EGFR mutations in primary and metastatic tumors and their response to TKIs, especially those with inconsistent measurement of EGFR mutation status in those sites. These studies could provide guidance for doctors to make informed decision in NSCLC treatment.

## Conclusions

Randomly chosen sample may reliably represent the type and ratio of EGFR mutations in primary tumors. EGFR mutation ratios in primary and metastatic tumors are different. If metastatic tumors are used for EGFR mutation detection, the sensitivity of the detection assay must be taken into consideration.

## Competing interests

The authors declare that they have no competing interests.

## Authors’ contributions

BW, KY and JZ carried out the DNA isolation. BW, YC, ZM, BD and YG performed real time PCR for quantification of EGFR mutation. BW and JM performed the statistical analysis. BW and JM designed the study and drafted the manuscript. All authors read and approved the final manuscript.
